# Loss of NDG-4 extends lifespan and stress resistance in *Caenorhabditis elegans*

**DOI:** 10.1111/acel.12165

**Published:** 2013-11-28

**Authors:** Jeanette Brejning, Steffen Nørgaard, Lone Schøler, Tine H Morthorst, Helle Jakobsen, Gordon J Lithgow, Louise T Jensen, Anders Olsen

**Affiliations:** 1 Department of Molecular Biology and Genetics, Aarhus UniversityGustav Wieds Vej 10C, Aarhus, 8000-DK, Denmark; 2 The Buck Institute for Research on Aging8001 Redwood Blvd, Novato, CA, 94945, USA

**Keywords:** aging, *C. elegans*, lipid transport, NDG-4, insulin signaling

## Abstract

NDG-4 is a predicted transmembrane acyltransferase protein that acts in the distribution of lipophilic factors. Consequently, *ndg-4* mutants lay eggs with a pale appearance due to lack of yolk, and they are resistant to sterility caused by dietary supplementation with the long-chain omega-6 polyunsaturated fatty acid dihommogamma-linolenic acid (DGLA). Two other proteins, NRF-5 and NRF-6, a homolog of a mammalian secreted lipid binding protein and a NDG-4 homolog, respectively, have previously been shown to function in the same lipid transport pathway. Here, we report that mutation of the NDG-4 protein results in increased organismal stress resistance and lifespan. When NDG-4 function and insulin/IGF-1 signaling are reduced simultaneously, maximum lifespan is increased almost fivefold. Thus, longevity conferred by mutation of *ndg-4* is partially overlapping with insulin signaling. The nuclear hormone receptor NHR-80 (HNF4 homolog) is required for longevity in germline less animals. We find that NHR-80 is also required for longevity of *ndg-4* mutants. Moreover, we find that *nrf-5* and *nrf-6* mutants also have extended lifespan and increased stress resistance, suggesting that altered lipid transport and metabolism play key roles in determining lifespan.

## Introduction

A growing number of genes and signaling pathways are being identified that determine lifespan in *Caenorhabditis elegans* (Lapierre & Hansen, 2012). With the detailed characterization of these genes, it is becoming increasingly clear that many of them function either in common signaling pathways or in overlapping molecular pathways that have extensive cross-talk. Several pathways have also been shown to function synergistically and additively in extending lifespan. Here, we report that the gene *ndg-4*, encoding a predicted transmembrane protein, is a novel gene acting additively with reduced insulin signaling to extend lifespan when inactivated.

The insulin/IGF-1-signaling (IIS) pathway is a central phosphatidylinositol-3-kinase (PI3-Kinase) signaling cascade determining *C. elegans* longevity, but it also influences many other biological processes such as development, dauer formation (an alternative hibernating larval stage), stress responses and metabolism (Kenyon, 2010). *daf-2* encodes the only insulin/IGF-1 receptor in *C. elegans* (Kenyon *et al*., 1993). Upon binding of insulin-like ligands DAF-2 phosphorylates the PI3-Kinase homolog AGE-1 (Morris *et al*., 1996). AGE-1 activates several other kinases, including AKT-1, SGK-1 and PDK-1, ultimately controlling the forkhead-family (FoxO) transcription factor, DAF-16. In its phosphorylated form, DAF-16 is sequestered in the cytoplasm and prevented from activating transcription or repression of target genes. Translocation of DAF-16 to the nucleus and subsequent activation is required for increased lifespan due to reduced IIS, and many DAF-16 target genes have been identified as modulators of lifespan (Lin *et al*., 1997; Ogg *et al*., 1997). In addition to IIS, DAF-16 also responds to signals from stress (Oh *et al*., 2005) and nutrient deprivation sensing cascades (Pan *et al*., 2007). Due to the importance of DAF-16 in such essential biological processes, a complex regulation of its activity has evolved involving expression of different isoforms (Kwon *et al*., 2010) as well as various DAF-16-binding proteins capable of regulating its activity (Cahill *et al*., 2001; Wang *et al*., 2006; Wolff *et al*., 2006; Li *et al*., 2008; Alam *et al*., 2010). Other proteins contribute to longevity of IIS mutants, such as the AMP-activated protein kinase α subunit AAK-2 (Apfeld *et al*., 2004), heat-shock factor HSF-1 (Hsu *et al*., 2003), the xenobiotic-response factor SKN-1/NRF (Tullet *et al*., 2008), and the ER unfolded-protein-response regulator XBP-1 (Henis-Korenblit *et al*., 2010). Interestingly, many of the genes regulated by these proteins are involved with responses to various types of stress, such as molecular chaperones, other heat-shock proteins, antioxidants, and detoxifying enzymes.

While there is growing evidence that both altered lipid metabolism and lipid signaling also play important roles in longevity, these are complex processes and the underlying molecular mechanisms are only beginning to be understood. In *C. elegans,* fat is stored in lipid droplets primarily in the intestine and in the hypodermal cells (Ashrafi, 2007). The long-lived *daf-2* mutants have increased fat content, and this is also a hallmark of the extremely long-lived dauers (Ogg *et al*., 1997). However, there is no direct correlation between increased fat accumulation and longevity, as not all mutants with increased fat accumulation are long lived, and likewise not all long-lived mutants have increased fat levels. In fact, the long-lived *eat-2* mutants have reduced fat stores (Brooks *et al*., 2009). Lipophilic hormonal signals from the *C. elegans* germ cells have an inhibitory effect on longevity and germline ablation increases lifespan (Hsin & Kenyon, 1999). The exact nature of this signal and how it is mediated is still largely unknown, but longevity due to reduced germline signaling requires a number of proteins including the nuclear hormone receptor DAF-12 (Hsin & Kenyon, 1999), DAF-16 and several others (McCormick *et al*., 2011). Genes involved in cellular checkpoint control are also involved in stress resistance and longevity (Bauer *et al*., 2005; Olsen *et al*., 2006; Arum & Johnson, 2007). As some checkpoint genes influence germ cell division, it is possible that they influence longevity via an altered germline signaling, but the underlying mechanisms still remain to be solved at the molecular level.

To identify novel genes determining longevity and stress resistance by mechanisms similar to checkpoint proteins, we performed a whole-genome RNAi screen for resistance to stalled replication forks using the chemotherapeutic drug hydroxyurea (HU; data not shown). One of the genes conferring resistance to HU after RNAi knock down was *ndg-4,* a gene encoding a protein containing an acyl transferease domain and 12 predicted transmembrane domains (Choy & Thomas, 1999). Prior to our study, *ndg-4* mutants were isolated in two different genetic screens. The first screen for resistance to *n*or *d*ihydro*g*uaiaretic acid (NDG) isolated the *ndg-4(lb108)* allele (Shreffler *et al*., 1995). NDG is a nonspecific lipoxygenase inhibitor that prevents synthesis of prostaglandins and leukotrienes. An additional allele *ndg-4(sa529)* was later isolated in a screen for fluoxetine resistance (Choy & Thomas, 1999). Fluoxetine is a serotonin reuptake inhibitor commonly used as an antidepressant (Prozac). Wild-type *C. elegans* worms exposed to fluoxetine contract their noses whereas a group of nose resistance to fluoxetine (Nrf) mutants, including *ndg-4,* do not exhibit this response (Choy & Thomas, 1999). Genetic analysis suggests that at least two independent pathways can cause resistance to fluoxetine (Choy *et al*., 2006). *ndg-4* may function in a pathway together with the Nrf mutants *nrf-5* and *nrf-6* (Choy & Thomas, 1999). *nrf-6* encodes an *ndg-4* homolog and *nrf-5* have homology to a mammalian secreted lipid binding protein. While *ndg-4* and *nrf-6* are expressed both in the intestine and in hypodermis, only their expression in the intestine is responsible for fluoxetine resistance (Choy & Thomas, 1999; Choy *et al*., 2006). *nrf-5* is also expressed in the intestine, but the NRF-5 protein is thought to be secreted into the pseudocoelomic fluid (Choy *et al*., 2006).

An independent study supports that *ndg-4*, *nrf-5* and *nrf-6* function in a common pathway in fat metabolism (Watts & Browse, 2006). Addition of the long-chain omega-6 polyunsaturated fatty acid dihommogamma-linolenic acid (DGLA, 20:3n-6) to the diet of the worms causes germ cell depletion and sterility in *C. elegans* (Watts & Browse, 2006). By contrast, DGLA does not interfere with the development and survival of the somatic gonadal cells. Interestingly, *ndg-4*, *nrf-5,* and *nrf-6* mutants are resistant to dietary addition of DGLA consistent with their gene products functioning in a common pathway transporting dietary lipids into the reproductive tract (Watts & Browse, 2006).

In this study, we report that *ndg-4*, *nrf-5,* and *nrf-6* are novel genes determining lifespan in *C. elegans*. Moreover, when NDG-4 function and insulin/IGF-1 signaling are reduced simultaneously, maximum lifespan is increased almost fivefold.

## Results

### *ndg-4* mutants are stress resistant and long lived

**Figure 1:**
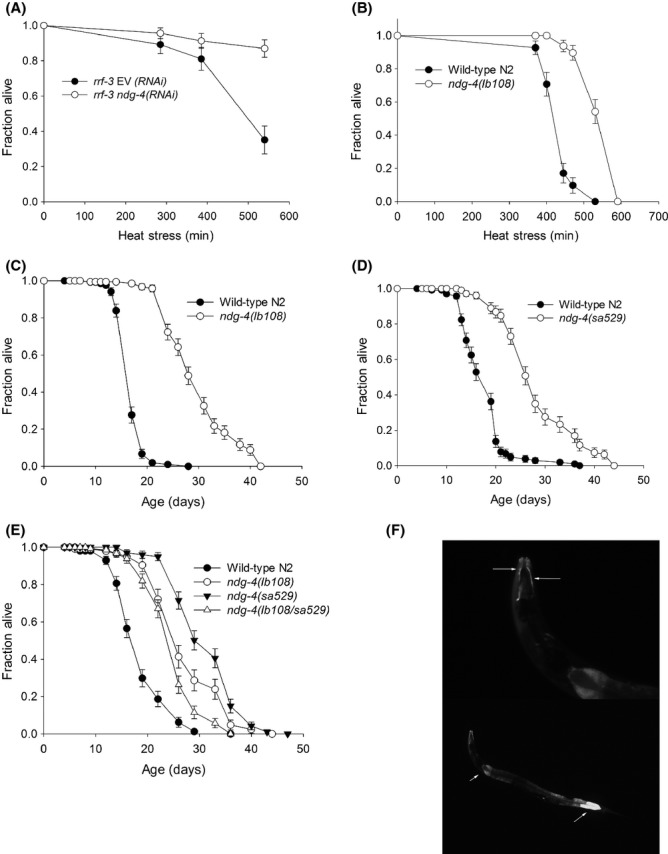
Loss of NDG-4 causes increased stress resistance and lifespan. (A) RNAi against *ndg-4* causes a significant increase in thermotolerance of *rrf-3(pk1426)* mutants. (B) *ndg-4(lb108)* mutants have significantly increased thermotolerance compared with wild-type N2 worms. (C) *ndg-4(lb108)* mutants have significantly increased lifespan compared with wild-type N2 worms. (D) *ndg-4(sa529)* mutants have significantly increased lifespan compared with wild-type N2 worms. (E) Lifespan of transheterozygous *ndg-4(sa529/lb108)* mutants*, ndg-4(lb108)* mutants, *ndg-4(sa529)* mutants and wild-type N2 worms. (F) Expression pattern of *ndg-4* shown by transgenic expression of the transcriptional reporter construct *Pndg-4::gfp*. Bottom: Strong *ndg-4* expression is seen in the intestine. Top: Enlargement showing *Pndg-4::gfp* expression in hypodermal cells in the nose region.

As RNAi against *ndg-4* caused resistance to replication stress (Fig. S1A), and *ndg-4* mutants had previously been shown to be resistant to NDG (Shreffler *et al*., 1995) and fluoxetine (Choy & Thomas, 1999), we hypothesized that *ndg-4* mutants had generally improved stress defense systems. To test whether *ndg-4* was involved with other types of stress resistance, we performed longitudinal thermotolerance assays. We found that *ndg-4* knockdown by RNAi caused significant resistance to heat stress at 35 °C compared with control worms fed empty vector (Fig. 1A; Table S1). In a parallel set of experiments, we examined the *ndg-4(lb108)* mutant strain originally isolated in a screen for resistance to NDG (Shreffler *et al*., 1995) and found that these mutants were also significantly thermotolerant compared with wild-type N2 worms (Fig. 1B; Table S1). We also observed that the *ndg-4(lb108)* mutants required an extra day to reach egg laying adulthood compared to wild-type N2 worms and that they laid pale eggs and had reduced brood size (data not shown), confirming previous observations (Choy & Thomas, 1999).

Because of the general relationship between longevity and stress resistance (Benedetti *et al*., 2008) the significant thermotolerance caused by reduced NDG-4 function prompted us to examine the lifespan of the *ndg-4(lb108)* mutants. We found that the *ndg-4(lb108)* mutants lived significantly longer than the wild-type N2 animals, with a mean lifespan of 29 ± 2 days compared with 17 ± 1 days for the wild-type N2 at 20 °C (Fig. 1C; Table S2).

Whereas RNAi against *ndg-4* resulted in thermotolerance (Fig. 1A; Table S1), no increase in lifespan was seen for worms treated from eggs with RNAi against *ndg-4* (the first generation; Table S2). Worms subjected to RNAi against *ndg-4* for two generations showed a small but significant increase in lifespan suggesting some maternal rescue (Fig. S1B; Table S2). It is not uncommon that reducing gene expression by RNAi does not phenocopy a mutation. However, to rule out the possibility of other unknown mutations in the genome being responsible for the observed phenotypes, we examined development and lifespan of mutants harboring another *ndg-4* allele, *sa529*. The *ndg-4(sa529)* mutant was originally isolated in a screen for resistance to fluoxetine (Choy & Thomas, 1999). We found that the *ndg-4(sa529)* mutation also conferred thermotolerance (Fig. S1C; Table S1) and longevity (Fig. 1D; Table S2) with lifespan increases similar to those seen for the *ndg-4(lb108)* allele (27 ± 2.0 days compared with 18 ± 0.5 for the wild-type N2). The *ndg-4(sa529)* mutants also developed slower than wild-type N2, had reduced brood sizes and laid pale eggs (data not shown). To confirm that the longevity was indeed conferred by mutation of *ndg-4* in both mutant strains, we examined the lifespan of trans-heterozygous *ndg-4(lb108/sa529)* mutants carrying one copy of each of the mutant *ndg-4* alleles. We confirmed that both alleles are recessive and found that these trans-heterozygous mutants also had significantly increased lifespan (25 ± 5 days compared with18 ± 5 for the wild-type N2; Fig. 1E).

### NDG-4 functions in the intestine and in hypodermal cells

To determine the temporal expression pattern of *ndg-4,* we generated a transcriptional reporter strain expressing green fluorescent protein (GFP) under the promoter of *ndg-4*. Strong GFP expression was seen in the intestine (Fig. 1F, lower panel) and in hypodermal cells, especially in the nose region (Fig. 1F, top panel), confirming previous observations (Choy & Thomas, 1999). This suggests that increased lifespan and stress resistance result from lack of *ndg-4* in these tissues. However, as our transgenic strain was made by microinjection, we cannot rule out possible germline expression of endogenous *ndg-4,* as expression of transgenes in the germline is often silenced.

### NDG-4 functions in a pathway partially overlapping with insulin signaling

**Figure 2:**
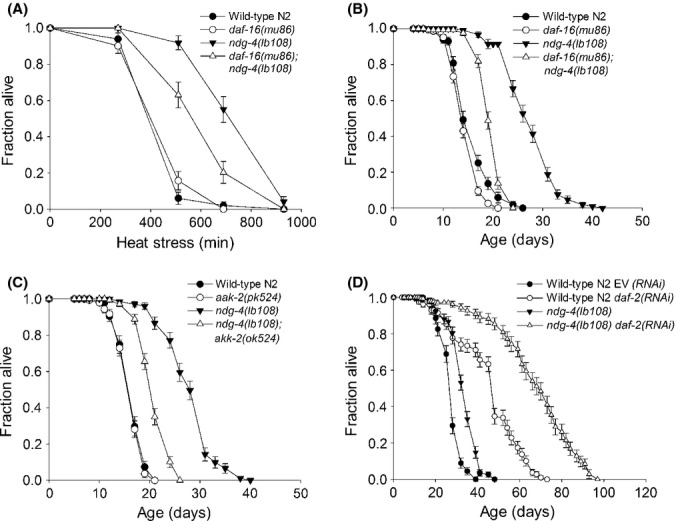
*ndg-4* and insulin-like signaling extend stress resistance and lifespan additively. (A) Thermotolerance of *ndg-4(lb108)* mutants is partially *daf-16-*dependent. (B) Lifespan extension of *ndg-4(lb018)* mutants is partially *daf-16-*dependent. (C) The lifespan extension of *ndg-4(lb108)* mutants is partially dependent on *aak-2*. (D) *daf-2* knockdown by RNAi increases the lifespan of *ndg-4* mutants by nearly 100%.

In terms of magnitude, the increases in lifespan conferred by the *ndg-4* alleles are comparable with those seen for the commonly used long-lived mutants, *age-1(hx546)* and *daf-2(e1370),* from the IIS pathway. Inactivation of the IIS pathway causes lifespan increase in a DAF-16-dependent manner (Kenyon *et al*., 1993). To establish whether stress resistance and lifespan extension by *ndg-4* mutation occur via the IIS pathway, we constructed *daf-16(mu86);ndg-4(lb108)* double mutants and examined their lifespan and thermotolerance. The *daf-16(mu86)* allele is thought to be null (Lin *et al*., 1997), and we found that lack of DAF*-*16 activity significantly reduced the thermotolerance and lifespan of *ndg-4(lb108)* mutants (Fig. 2A,B; Tables S1 and S2). However, the *daf-16(mu86);ndg-4(lb108)* double mutants were still significantly more stress resistant and longer lived than *daf-16 (mu86)* single mutants. The *daf-16(mu86)* mutation did not suppress the pale egg phenotype or the slow growth phenotype of the *ndg-4* mutants (data not shown). Therefore, we conclude that NDG-4 is functioning in a manner partially overlapping with the IIS pathway.

To further study involvement of IIS signaling, we investigated dependence on the AMP-activated protein kinase α subunit *aak-2*, as AAK-2 is also necessary for lifespan extension of *daf-2* mutants. AAK-2 responds to stressors, energy levels and insulin signaling. AAK-2 has been proposed to act in parallel to DAF-16 to extend lifespan in *daf-2* mutants (Apfeld *et al*., 2004). Therefore, we predicted that AAK-2 would be necessary for the increased lifespan of *ndg-4* mutants. To test this, we constructed an *aak-2(ok524);ndg-4(lb108)* double mutant and performed lifespan analysis. The *aak-2(ok524)* allele significantly, but not completely, reduced the lifespan of *ndg-4* mutants (Fig. 2C), supporting the notion that the longevity of *ndg-4* mutants is partially overlapping with IIS. The reduction in lifespan was similar to that seen by introduction of the *daf-16(mu86)* mutation. The *aak-2(ok524);ndg-4(lb108)* double mutants had a mean lifespan of 20.9 ± 2.9 days compared to 33.0 ± 6.8 days for *ndg-4(lb108)* mutants.

As a major part of the lifespan increase in *ndg-4* mutants could be attributed to IIS dependent mechanisms, we predicted that RNAi against *daf-2* would only result in a modest increase in the lifespan of *ndg-4* mutants compared with RNAi against *daf-2* in a wild-type N2 background. Intriguingly, we observed an additive effect on lifespan when *ndg-4* mutants were treated with RNAi against *daf-2* (Fig. 2D; Table S2). The longest lived *ndg-4;daf-2(RNAi)* worms had a maximum lifespan of close to 100 days. The lifespan increase in *ndg-4* mutants due to RNAi against *daf-2* was fully dependent on *daf-16* (Table S2). Other additive effects with insulin signaling with regards to longevity have previously been reported for other genes and pathways, including for example reduced translation (Pan *et al*., 2007) and signals from the germline (Spanier *et al*., 2010).

### Inactivation of *chk-1* does not increase stress resistance of *ndg-4* mutants

As *ndg-4(RNAi)* worms were able to grow to adulthood in the presence of the chemotherapeutic drug HU (Fig. S1A), we speculated that *ndg-4* mutants might be checkpoint deficient and that this could be responsible for their stress resistance and longevity. Checkpoint proteins secure proper cell division and play key roles in regulating apoptosis. Using the CED-1::GFP marker (Zhou *et al*., 2001), we found that *ndg-4(lb108)* mutants had a slightly elevated number of apoptotic cells per gonad arm compared with the wild-type N2 controls (Mean 2.14, *n* = 51 compared with 0.18, *n* = 60, *P* < 0.05 Student’s *t*-test). Following exposure to DNA damaging ionizing radiation we observed a similar increase in the number of apoptotic cells per gonad arm in *ndg-4(lb108)* mutants (6.47, *n* = 30) and wild-type N2 controls (7.00, *n* = 27). Thus, *ndg-4* is not required for mounting a normal apoptotic checkpoint response to DNA damage (Fig. S1C).

**Figure 3:**
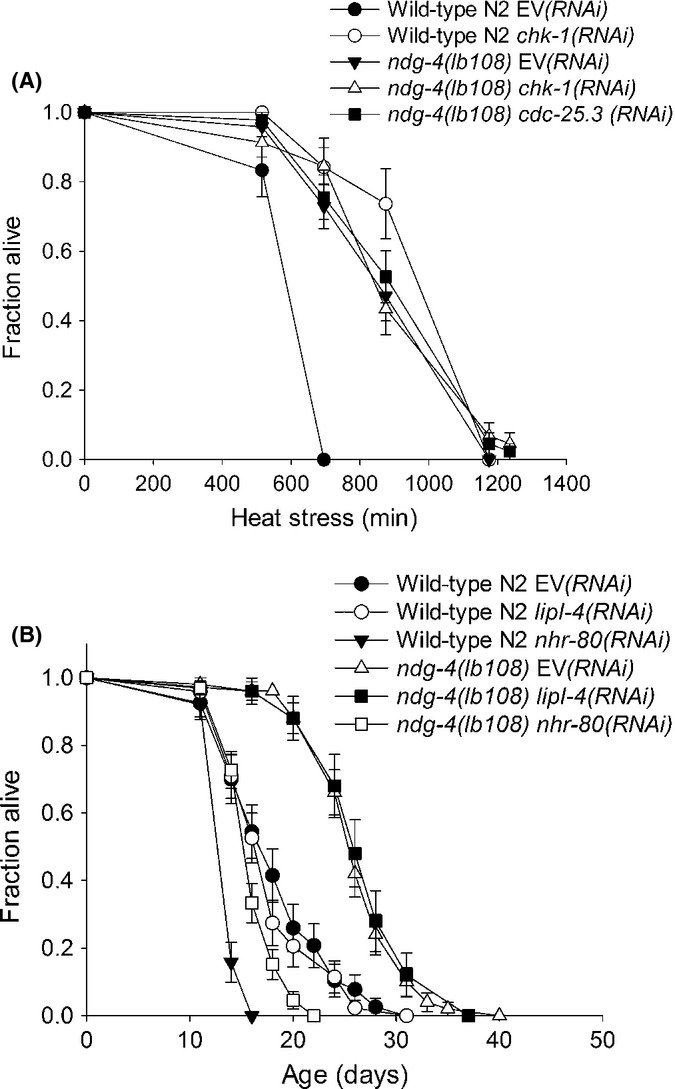
HNR-80 is required for longevity of *ndg-4* mutants. (A) Increased thermotolerance is observed when the S-M checkpoint genes *chk-1* and *cdc-25.3* are RNAi inactivated in wild-type N2 worms. The thermotolerance of *ndg-4* is not further increased following RNAi inactivation of *chk-1* and *cdc-25.3*. (B) RNAi against *nhr-80* completely abolishes longevity of *ndg-4* mutants whereas RNAi against *lipl-4* has no effect.

The serine threonine kinase CHK-1 is required for the DNA damage and S-M checkpoints and is necessary for germline development (Kalogeropoulos *et al*., 2004). *chk-1* knockdown by RNAi confers increased thermotolerance and lifespan as does knockdown of the downstream phosphatase *cdc-25* (Olsen *et al*., 2006; de Lencastre *et al*., 2010). To further test a possible involvement of checkpoint proteins in the longevity of the *ndg-4* mutants, we examined the effect of inactivating *chk-1*. RNAi against *chk-1* significantly increased the thermotolerance of wild-type worms but had no or limited effect on *ndg-4(lb108)* mutants (Fig. 3A and Table S1). The same phenomenon was observed by knockdown of *cdc-25.3* (Fig. 3A; Table S1). These data are suggesting that stress resistance in *ndg-4* mutants and checkpoint mutants may be conferred by the same mechanism. Although not completely resolved at the molecular level, it has been suggested that the increase in longevity following knockdown of checkpoint proteins is due to lack of germline signaling (de Lencastre *et al*., 2010).

### *ndg-4* mutants are long lived due to altered germline signaling

As lifespan extension following germline removal acts synergistically with the IIS pathway (Hsin & Kenyon, 1999), and as RNAi against *chk-1* (germline less) did not increase thermotolerance of *ndg-4* mutant worms (Fig 3A; Table S1), we next investigated if *ndg-4* mutants had increased lifespan due to altered germline signaling. The nuclear hormone receptor NHR-80 (HNF4 homolog) has recently been shown to be required specifically for longevity in germline less animals but not for other life extending pathways such as IIS and dietary restriction (Goudeau *et al*., 2011). NHR-80 regulates oleic acid synthesis via the desaturases *fat-5*, *fat-6,* and *fat-7* and links fatty acid synthesis and longevity resulting from germline removal (Lapierre & Hansen, 2012). We found that RNAi inactivation of *nhr-80* completely abolished the increased lifespan of the *ndg-4* mutants (Fig. 3B; Table S1). This is consistent with *ndg-4* mutants being long lived due to altered germline signaling. However, it should be noted that in our experiments RNAi against *nhr-80* also significantly decreased the lifespan of wild-type N2 control animals, suggesting that it may not uniquely be required for longevity due to altered germline signaling as previously reported (Goudeau *et al*., 2011).

Several independent pathways have been described as regulators of longevity in germline less animals. The lipase *lipl-4* (K04A8.4) is induced in germline less animals in a *daf-16*-dependent manner (Wang *et al*., 2008; Lapierre *et al*., 2012) in contrast to *nhr-80,* which acts independently of *daf-16* (Goudeau *et al*., 2011). We did not observe any significant decrease in the lifespan of *ndg-4* mutants following RNAi against *lipl-4* (Fig. 3B; Table S2). Hence, our data support that *nhr-80* and *lipl-4* influence germline signals via separate pathways and that *ndg-4* is part of the former pathway.

### *nrf-5* and *nrf-6* also determine stress resistance and lifespan

In addition to *ndg-4,* mutations in six other genes named *nrf-1* to *-6* were reported to confer the Nrf phenotype (Choy & Thomas, 1999). Of these, *nrf-5, nrf-6,* and *ndg-4* define a subclass of Nrf mutants sharing an additional second phenotype of producing pale eggs with fewer yolk granules. Members of this subclass also accumulate large globules of yolk in the pseudocoelomic space, develop slower than wild-type N2 worms and produce a large percentage of dead embryos. The genes in this subclass have been suggested to function in the same pathway or complex to confer fluoxetine-induced nose contraction (Choy & Thomas, 1999). None of the members of the other subclass comprised by *nrf-1* to *nrf-4* were reported to produce pale eggs (Choy & Thomas, 1999). To determine whether stress resistance and longevity might be correlated with the pale egg or Nrf phenotype, we examined lifespan and thermotolerance of these mutants. We found that *nrf-5* and *nrf-6* mutants (pale egg) were long-lived and that *nrf-1*, *nrf-2, nrf-3,* and *nrf-4* mutants (Nrf but not pale egg) had wild-type or shortened lifespan (Table S2). In agreement with this, we found that *nrf-1, nrf-5,* and *nrf-6* mutants were more thermotolerant than wild-type worms, whereas *nrf-2, nrf-3,* and *nrf-4* mutants had wild-type thermotolerance (Table S1). Thus, with *nrf-1* being the exception, it seems that longevity is associated with the pale egg phenotype. We cannot rule out that the lifespan of the *Nrf* mutants would change upon backcrossing, but our data show that the Nrf phenotype *per se* is likely not linked to longevity or stress resistance.

### *ndg-4* and *nrf-5* might function in a common pathway to determine stress resistance and lifespan

**Figure 4:**
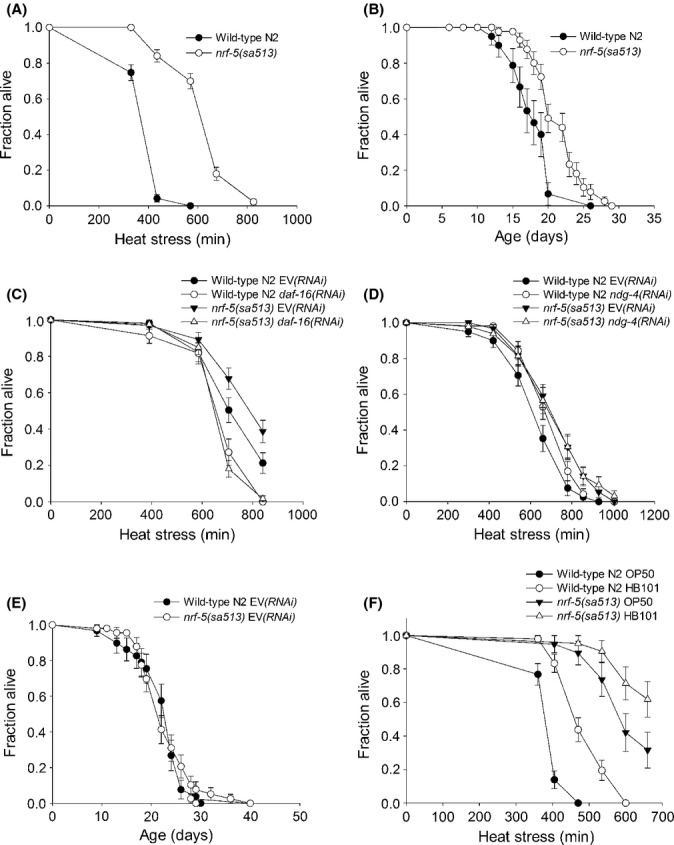
*nrf-5* mutants are stress resistant and long-lived. (A) *nrf-5(sa513)* mutants are significantly thermotolerant compared with and wild-type N2 worms. (B) *nrf-5(sa513)* mutants are significantly longer lived than wild-type N2 worms at 20 °C. (C) *nrf-5(sa513)* mutants are thermotolerant when fed HT115 empty vector RNAi bacteria and this is dependent on *daf-16*. (D) Thermotolerance of *nrf-5(sa513)* mutants is not further increased by RNAi against *ndg-4. (E) nrf-5(sa513)* mutants are not long lived when fed HT115 empty vector RNAi bacteria. (F) Feeding a HB101 diet cause significant thermotolerance of wild-type N2 worms and *nrf-5* mutants compared with feeding an OP50 diet.

We decided to investigate *nrf-5* mutants in more detail because of reports placing *ndg-4* and *nrf-5* in the same genetic pathway or complex (Choy *et al*., 2006; Watts & Browse, 2006). In addition to the pale egg phenotype, we noticed that the *ndg-4* and *nrf-5* mutants also have slow development in common (data not shown). We found that the *nrf-5* mutants had a significant increase in both thermotolerance (Fig. 4A) and lifespan (Fig. 4B) with a mean lifespan of 20.8 ± 3 days compared with 17.8 ± 3 days for the wild-type N2. The increase in lifespan observed for *nrf-5* mutants was smaller than that observed for *ndg-4* mutants, whereas their levels of thermotolerance were comparable (Figs 1B and 4A). Consistent with *ndg-4* and *nrf-5* functioning in a common pathway, we found that thermotolerance of the *nrf-5* mutants were also dependent on the FoxO transcription factor DAF-16 (Fig. 4C). To further investigate whether *ndg-4* and *nrf-5* might function in a common pathway in terms of thermotolerance, we inactivated *ndg-4* using RNAi in an *nrf-5* mutant background. We found that RNAi against *ndg-4* did not further increase thermotolerance of *nrf-5* mutants (Fig. 4D). In terms of fluoxetine resistance and DGLA transport, *ndg-4* and *nrf-5* have been placed in a common pathway (Choy *et al*., 2006; Watts & Browse, 2006). Our data are consistent with *ndg-4* and *nrf-5* also functioning in a common pathway in terms of longevity and stress resistance. However, protein interaction studies need to be performed to establish if they actually function in a protein complex as previously suggested (Watts & Browse, 2006).

### The bacterial diet influences stress resistance

We also investigated the effect of RNAi against *nrf-5* but found that it did not cause increased stress resistance or lifespan; despite a knockdown efficiency of nearly 98% (data not shown). The bacterial diet affects fat stores and lifespan in *C. elegans*. Specifically, feeding with HT115 or HB101 bacteria instead of OP50 leads to reduced fat stores and increased lifespan (Brooks *et al*., 2009). Intriguingly, we observed that the longevity of the *nrf-5* mutant strain was either abolished or significantly reduced when the mutants were fed HT115 RNAi feeding bacteria rather than the normal food strain OP50 (Fig. 4E; Table S2). We speculated that perhaps *nrf-5* mutants would only be long-lived when fed a diet not leading to reduced fat stores. To test this, we investigated feeding with HB101 bacteria. We found that the HB101 diet accelerated development of both wild-type N2 and *nrf-5* mutants (data not shown), but that *nrf-5* mutants were still long-lived when fed a HB101 diet (Table S2), showing that their longevity does not depend on a diet that leads to reduced fat stores. Supporting this, we found that *nrf-5* mutants fed either HT115 or HB101 bacteria were significantly thermotolerant compared with wild-type N2 (Fig. 4A,C,D,F; Table S1). Moreover, feeding with HB101 bacteria significantly increased thermotolerance of wild-type N2 worms compared to feeding an OP50 diet (Fig. 4F). Thus, in agreement with previous studies showing that the bacterial diet can increase lifespan (Brooks *et al*., 2009), our data show that also stress resistance can be influenced by the bacterial diet.

## Discussion

In this study, we describe how mutation of the gene *ndg-4* leads to significant increases in lifespan and stress resistance. The increases in lifespan observed in *ndg-4* mutants are more than 50% compared to wild-type controls. These phenotypes are partially dependent on *daf-16,* and simultaneous reduction of insulin signaling and *ndg-4* leads to nearly a doubling of the already long lifespan with maximum lifespan reaching 100 days. The *ndg-4* gene encodes a transmembrane protein with predicted acyltransferase activity that has not previously been described as having a role in lifespan determination in *C. elegans*. Earlier studies have shown that mutation of *ndg-4* confers resistance to fluoxetine (nose resistant to fluoxetine Nrf phenotype) and NDG. We find that two other Nrf mutants, *nrf-5,* and *nrf-6* encoding a putative lipid-binding protein and a *ndg-4* homolog, respectively, also have increased stress resistance and lifespan, but that other Nrf mutants do not have this phenotype. It has previously been suggested that *ndg-4* and *nrf-5* might function in a common pathway or even in a complex in determining fluoxetine resistance, and our data support this notion also in terms of lifespan. We generally observe that the animals live longer when fed HT115 bacteria instead of OP50. These results are consistent with a previous study showing that feeding *C. elegans* HT115 or HB101 bacteria rather than OP50 increases mean lifespan and changes the fatty acid content (Brooks *et al*., 2009). Interestingly, the *nrf-5* mutants fed HT115 bacteria did not have increased lifespan. Thus, the underlying mechanism for longevity in *nrf-5* mutants could be the same mechanism causing longevity due to a HT115 diet. In contrast, feeding on a HB101 diet increased the lifespan of *nrf-5* mutants. Stress resistance and increased lifespan are tightly linked in *C. elegans*. Supporting this notion, we find that feeding a HT115 or HB101 diet increases thermotolerance compared to feeding an OP50 diet. However, in contrast to lifespan, we find that feeding a HT115 or HB101 diet also increases thermotolerance in *nrf-5* mutants. This suggests that a HT115 and a HB101 diet can confer thermotolerance and longevity through two distinct pathways.

There is growing evidence that fat metabolism and lipid-signaling might play important roles in longevity (Lapierre & Hansen, 2012). Given the previously described roles of *ndg-4* and *nrf-5* in fatty acid and yolk transport (Watts & Browse, 2006) and that *ndg-4* longevity depends on *nhr-80,* we suggest that altered fat metabolism and/or germline signaling is responsible for the longevity and stress resistance of these mutants. This is consistent with the observation that only Nrf mutants with defective yolk transport (pale eggs) were found to be long-lived.

Hormonal signals from the germline are involved in lifespan determination and linked to lipid metabolism. One possibility is that in *ndg-4* and *nrf-5* mutants such hormonal signal is either not produced due to impaired delivery of a required substrate, or that the hormone does not correctly reach its intended target after it has been synthesized. Lifespan extension due to germline ablation requires several proteins including the FoxO transcription factor DAF-16 and the nuclear hormone receptor DAF-12 (Hsin & Kenyon, 1999). The increases in lifespan and stress resistance of the *ndg-4* mutants are only partially dependent on DAF-16. Hence, a signal identical to that in germline ablated animals cannot completely explain their longevity because longevity due to germline ablation is completely suppressed by mutation of *daf-16*. However, it is possible that more than one signal from the germline can influence lifespan and that such signals will elicit different molecular responses. This view is supported by the recent finding that overexpression of NHR-80 can extend the lifespan of germline ablated animals in a DAF-16 independent manner, but it requires the nuclear hormone receptor DAF-12 (Goudeau *et al*., 2011).

The synergistic effect observed between *ndg-4* and reduced insulin signaling is striking when considering the already impressive lifespan observed due to reduced insulin signaling. However, there are other examples of similar synergistic effects between reduced insulin signaling and other longevity pathways such as reduced TOR signaling/translation (Hansen *et al*., 2007; Pan *et al*., 2007; Robida-Stubbs *et al*., 2012) and altered germline signaling (Arantes-Oliveira *et al*., 2003). Germline ablation also significantly increases the lifespan of insulin-signaling mutants (Hsin & Kenyon, 1999) and altered signaling from the germline to the intestine is responsible for the long lifespan of double mutants between *daf-2* and the intestinal di- and tripeptide transporter *pept-1* (Spanier *et al*., 2010). Thus, the synergistic effect between an *ndg-4* mutation and reduced insulin signaling is consistent with altered lipid signaling being responsible for the longevity. In *C. elegans,* fat is stored in lipid droplets primarily in the intestine and in the hypodermal cells (Ashrafi, 2007). Thus, a role of NDG-4 in lipid metabolism/signaling is supported by *ndg-4* being expressed in the intestine, which has also been suggested in previous studies (Choy *et al*., 2006; Watts & Browse, 2006).

The NDG-4 protein contains an acyltransferase domain (Choy & Thomas, 1999). Based upon homology, there are several putative acyl transferases in *C. elegans,* and as these are not redundant to *ndg-4* in terms of longevity, it is likely that they have different substrates. It is important to identify such substrates of NDG-4 and determine if they play conserved roles in longevity in other organisms. In this regard, it is interesting to note that in *Drosophila melanogaster* mutation of the NDG-4 homolog *drop-dead* (drd gene) causes accelerated aging and abrupt and early onset of death (Rogina *et al*., 1997). The *drop-dead* mutants have defects in movement of food in the gut (Peller *et al*., 2009), but they also suffer from severe neurodegeneration (Blumenthal, 2008). The neuronal damage is possibly associated with hypoxia in the brain (Kim *et al*., 2012). It is possible that these severe developmental defects mask an otherwise positive effect on longevity. These studies further stress the importance of identifying targets of NDG-4 because they could uncover novel mechanisms determining longevity.

## Experimental procedures

### *Caenorhabditis elegans* strains and culture conditions

Strains of *C. elegans* were cultured using standard protocols. The N2 Bristol strain was used as wild-type. For most experiments with RNAi, the RNAi hypersensitive strain NL2099 *rrf-3(pk1426)* II was used. The strain MP108 *ndg-4 (lb108)* III was backcrossed twice to our wild-type N2 strain to generate the strain OLS11 used in all *ndg-4* experiments. We specifically ensured that a *bus-1* mutation was eliminated using PCR. The strain JT513 *nrf-5(sa513)* V was backcrossed 2 and 3 times to our wild-type N2 strain following the slow development and pale egg phenotypes to generate the strains OLS53 and OLS51, respectively. We choose these phenotypes because they have previously been rescued by transgenic expression of the *nrf-5* gene (Choy *et al*., 2006). Furthermore, to test that the slow development and low embryonic survival of the *nrf-5* mutant was indeed due to the *nrf-5(sa513)* V mutation we took advantage of the mutant strain RB786 containing a large deletion in the gene *stdh-1* located 0.09 cm away from *nrf-5* on chromosome V. As the genes are located within such close proximity, the possibility of cross-over between them is minimal. We next analyzed the F2 progeny from a cross between *nrf-5* hermaphrodites and *stdh-1* males for slow development and the presence or absence of the *stdh-1* deletion. All progeny that were homozygous for the *nrf-5* mutation had slow development and low embryonic survival, as did the F3 and F4 progeny. All F2 progeny that was homozygous or heterozygous for the *stdh-1* deletion had wild-type development and embryonic survival, as did the F3 and F4 progeny confirming that *nrf-5* is indeed responsible for the phenotype. The *ndg-4(sa529)* III mutant was backcrossed once to our wild-type N2 to ensure the mutation was recessive before generating the transheterozygote *ndg-4* mutants. JT524 *nrf-1(sa524)* III*,* JT366 *nrf-2/vhp-1(sa366)* II*,* JT363 *nrf-3(sa363)* IV, JT528 *nrf-4(sa528)* I, and JT525 *nrf-6(sa525)* II were not backcrossed because they showed no phenotypes relevant for this study. The slow growth and the pale egg phenotypes previously shown to be rescued by transgenic expression of *ndg-4* (Choy & Thomas, 1999) were used to follow the *ndg-4(lb108)* mutation in all crosses. PCR was used to follow the *daf-16(mu86)* deletion. The transgenic strain OLS22 expressing GFP under control of the *ndg-4* promotor (p*ndg-4*::GFP) was generated by means of microinjection in DP38 *unc-119* mutants. The pENTRY *ndg-4* promotor clone was obtained from Open Biosystems and inserted in the pDEST DDO4 using a Gateway LR reaction. Several independent lines showed similar expression patterns.

### RNAi treatment

RNAi feeding bacteria were obtained from Open Biosystems except bacteria expressing *daf-2* dsRNA, which was a kind gift from Dr. Andrew Dillin, and bacteria expressing *lipl-4* and *nhr-80* dsRNA were kind gifts from Dr. Nils Færgeman. The bacteria expressing *nrf-5* dsRNA were generated as described (Timmons, 2006). RNAi bacteria were maintained on Luria Broth (LB) plates with 100 μg mL^−1^ ampicillin and 12.5 μg mL^−1^ tetracycline. For experiments, RNAi bacteria were grown at 37 °C overnight in LB with 100 μg mL^−1^ ampicillin. The next day cultures were spotted onto RNAi plates consisting of NGM agar with 100 μg mL^−1^ ampicillin and 1 mM isopropyl 1-thio-β-D-galactopyranoside. After incubation at room temperature for at least 1 day, eggs were placed on spotted RNAi plates. Control animals were fed bacteria carrying an empty L4440 vector. For RNAi knockdown of *chk-1*, *cdc-25.3,* and *ndg-4* (except where otherwise stated), experiments were performed in the second generation of growth on RNAi bacteria.

### Lifespan analysis

Lifespan assays were performed as previously described (Olsen *et al*., 2006). Briefly, during the reproductive period, worms were transferred to fresh plates every day or every other day. After the reproductive period, worms were transferred every 3–5 days. Survival was scored as touch provoked movement. Bagged or exploded worms were scored as lost and censored. Survival data were analyzed using Graphpad Prism version 5 (GraphPad Software, Inc., La Jolla, CA, USA). Survival was plotted as surviving fraction ± SEM.

### Thermotolerance assays

Longitudinal thermotolerance assays were performed as previously described (Olsen *et al*., 2006). Briefly, 3 cm NGM plates with each 25–30 synchronous four- or 5-day-old adults were shifted from their normal growth temperature to 35 °C and survival scored as indicated as touch provoked movement. Data were analyzed and plotted as described for lifespan assays.

### Germline apoptosis and ionizing radiation

*The ndg-4;ced-1::gfp* strain was made by crossing MD701 *ced-1::gfp* with OLS11 *ndg-4(lb108)*. Worms were grown at 25 °C on OP50 and scored for apoptotic cells 24 h post the L4 stage by mounting them i S-basal [0.1 m NaCl, 0.05 m H_2_PO_4_ (pH 6)] on slides with 2% agarose. Slides were sealed with nail polish, and the worms were immobilized by a brief exposure to heat. For ionizing radiation, the worms were irradiated at the L4 stage with 90 Gy and apoptotic cells in the death zone of the germline were scored 24 h later.

## Supporting Information

**Fig. S1.** (A) RNAi against *ndg-4* confers resistance to hydroxyurea at a concentration (10.16 mm) where control animals arrest their development. Animals were cultured for 4 days at 20 °C before their development was scored. (B) RNAi against *ndg-4* causes a small but significant (*P* < 0.05) increase in lifespan of *rrf-3(pk1426)* mutants when kept on RNAi bacteria for two generations compared to controls. (C) *ndg-4(sa529)* mutants have significantly increased (*P* < 0.0007) thermotolerance compared to wild-type N2 worms. (D) Expression of the apoptosis marker CED-1:.GFP in wild-type N2 animals and *ndg-4(lb108)* mutants was used to quantify apoptosis in the death zone of the germline. *ndg-4* mutants have significantly (*P* < 0.0001) higher levels of apoptotic cells (2.2 ± 0.1, *n* = 40) compared to controls (0.6 ± 0.5, *n* = 40). Error bars are standard error of mean.

**Table S1** Summary of longitudinal thermotolerance assays.

**Table S2** Summary of lifespan assays at 20 °C except experiment S1B^#^, which was at 25 °C.
