# Supplementary material for: Loss of NDG-4 extends lifespan and stress resistance in Caenorhabditis elegans
Source: Aging Cell. 2013 Nov 28;13(1):156–64. doi: 10.1111/acel.12165 (PMC3919970; doi:10.1111/acel.12165)
Supplement: Supplementary file 1 — Fig. S1. (A) RNAi against ndg-4 confers resistance to hydroxyurea at a concentration (10.16 mm) where control animals arrest their development. Animals were cultured for 4 days at 20 °C before their development was scored. (B) RNAi against ndg-4 causes a small but significant (P < 0.05) increase in lifespan of rrf-3(pk1426) mutants when kept on RNAi bacteria for two generations compared to controls. (C) ndg-4(sa529) mutants have significantly increased (P < 0.0007) thermotolerance compared to wild-type N2 worms. (D) Expression of the apoptosis marker CED-1:.GFP in wild-type N2 animals and ndg-4(lb108) mutants was used to quantify apoptosis in the death zone of the germline. ndg-4 mutants have significantly (P < 0.0001) higher levels of apoptotic cells (2.2 ± 0.1, n = 40) compared to controls (0.6 ± 0.5, n = 40). Error bars are standard error of mean. [file acel0013-0156-sd1.docx]

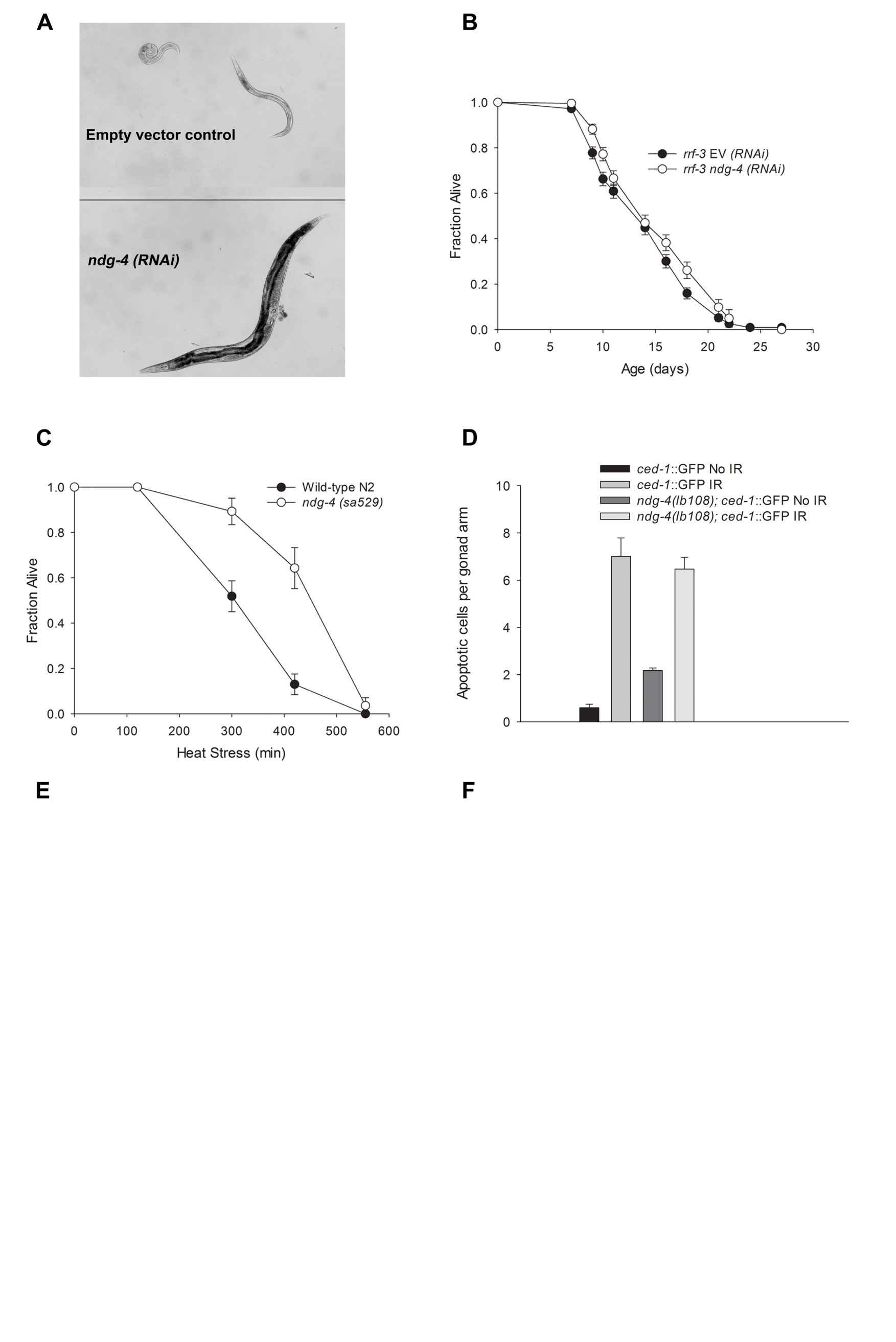


**Supplemental Fig. S1**

**A.** RNAi against *ndg-4* confers resistance to hydroxyurea at a concentration (10.16 mM) where control animals arrest their development. Animals were cultured for 4 days at 20°C before their development was scored. **B**. RNAi against *ndg-4* causes a small but significant (P<0.05) increase in lifespan of *rrf-3(pk1426)* mutants when kept on RNAi bacteria for two generations compared to controls. **C**. *ndg-4(sa529)* mutants have significantly increased (P<0.0007) thermotolerance compared to wild-type N2 worms. **D**. Expression of the apoptosis marker CED-1:.GFP in wild-type N2 animals and *ndg-4(lb108)* mutants was used to quantify apoptosis in the death zone of the germline. *ndg-4* mutants have significantly (P<0.0001) higher levels of apoptotic cells (2.2 ± 0.1, n=40) compared to controls (0.6 ± 0.5, n=40). Error bars are standard error of mean.
