# Supplementary material for: Loss of NDG-4 extends lifespan and stress resistance in Caenorhabditis elegans
Source: Aging Cell. 2013 Nov 28;13(1):156–64. doi: 10.1111/acel.12165 (PMC3919970; doi:10.1111/acel.12165)
Supplement: Supplementary file 2 — Table S1 Summary of longitudinal thermotolerance assays. [file acel0013-0156-sd2.docx]

| Strain | Mean Survival Minutes ±SD (deaths recorded) |  | P-value  Log-rank test |  |
| --- | --- | --- | --- | --- |
| NL2099 *rrf-3(pk1426)* *EV(RNAi)* | > 729 (35) |  |  |  |
| NL2099 *rrf-3(pk1426)* *ndg-4(RNAi)* | > 760 (19) |  | <0.012 |  |
|  |  |  |  |  |
| NL2099 *rrf-3(pk1426)* *EV(RNAi)* | > 500 (22) |  |  |  |
| NL2099 *rrf-3(pk1426)* *ndg-4(RNAi)* | > 523 (6) |  | < 0.0001 |  |
|  |  |  |  |  |
| Wild-type N2 | 380 ± 88 (54) |  |  |  |
| JT529 *ndg-4(sa529)* | > 494 (27) |  | < 0.0007 |  |
|  |  |  |  |  |
| Wild-type N2 | 511 ± 92 (50) |  |  |  |
| CF1038 *daf-16(mu86)* | 511 ± 102 (50) |  |  |  |
| OLS11 *ndg-4(lb108)* | > 808 (47) |  | < 0.0001 |  |
| OLS15 *ndg-4(lb108); daf-16(mu86)* | 658 ± 147 (43) |  | < 0.0001 |  |
|  |  |  |  |  |
| Wild-type N2 *EV(RNAi)* | 665 ± 69 (24) |  |  |  |
| N2 wildtype *chk-1(RNAi)* | 1068 ± 190 (19) |  | < 0.0001 |  |
| OLS11 *ndg-4(lb108) EV(RNAi)* | 953 ± 222 (46) |  |  |  |
| OLS11 *ndg-4(lb108) chk-1(RNAi)* | > 964 ± 221 (42) |  | ns* |  |
| OLS11 *ndg-4(lb108) cdc25.3(RNAi)* | > 985 (43) |  | ns* |  |
|  |  |  |  |  |
| Wild-type N2 *EV(RNAi)* | 622 ± 124 (22) |  |  |  |
| N2 wildtype *chk-1(RNAi)* | > 846 (12) |  | < 0.0001 |  |
| OLS11 *ndg-4(lb108)* *EV(RNAi)* | > 782 (15) |  |  |  |
| OLS11 *ndg-4(lb108)* *chk-1(RNAi)* | > 756 (17) |  | ns |  |
| OLS11 *ndg-4(lb108)* *cdc-25.3(RNAi)* | > 844 (30) |  | ns* |  |
|  |  |  |  |  |
| Wild-type N2 *EV(RNAi)* | 456 ± 77 (25) |  |  |  |
| N2 wildtype *chk-1(RNAi)* | > 782 (16) |  | < 0.0001 |  |
| OLS11 *ndg-4(lb108) EV(RNAi)* | > 957 (48) |  |  |  |
| OLS11 *ndg-4(lb108) chk-1(RNAi)* | > 1042 (26) |  | < 0.0002* |  |
| OLS11 *ndg-4(lb108) cdc25.3(RNAi)* | > 1034 (34) |  | < 0.0002* |  |
|  |  |  |  |  |
| Wild-type N2 *EV(RNAi)* | 720 ± 192 (22) |  |  |  |
| N2 wildtype *chk-1(RNAi)* | > 1023 (12) |  | < 0.0001 |  |
| OLS11 *ndg-4(lb108)* *EV(RNAi)* | > 1017 (15) |  |  |  |
| OLS11 *ndg-4(lb108)* *chk-1(RNAi)* | > 933 (11) |  | ns * |  |
| OLS11 *ndg-4(lb108)* *cdc-25.3(RNAi)* | > 1024 (12) |  | ns * |  |
|  |  |  |  |  |
| Wild-type N2 *EV(RNAi)* | 926 ± 149 (22) |  |  |  |
| N2 wildtype *chk-1(RNAi)* | > 1035 (17) |  | 0.0004* |  |
| OLS11 *ndg-4(lb108)* *EV(RNAi)* | > 1058 (36) |  |  |  |
| OLS11 *ndg-4(lb108)* *chk-1(RNAi)* | > 1067 (32) |  | ns* |  |
| OLS11 *ndg-4(lb108)* *cdc-25.3(RNAi)* | > 1059 (36) |  | ns |  |
|  |  |  |  |  |
| Wild-type N2 | 380 ± 88 (54) |  |  |  |
| JT524 *nrf-1(sa524)* | > 431 ± 113 (53) |  | 0.0042* |  |
| JT366 *nrf-2(sa366)* | 387 ± 78 (56) |  | ns* |  |
| JT363 *nrf-3(sa363)* | 342 ± 89 (51) |  | ns* |  |
| JT528 *nrf-4(sa528)* | 394 ± 134 (58) |  | ns* |  |
| JT513 *nrf-5(sa513)* | > 504 ± 78 (55) |  | <0.0007* |  |
| JT525 *nrf-6(sa525)* | 468 ± 81 (55) |  | <0.0007* |  |
|  |  |  |  |  |
| Wild-type N2 | > 546 (53) |  |  |  |
| JT524 *nrf-1(sa524)* | > 546 (47) |  | ns* |  |
| JT366 *nrf-2(sa366)* | > 524 (47) |  | ns* |  |
| JT363 *nrf-3(sa363)* | > 573 (29) |  | ns* |  |
| JT528 *nrf-4(sa528)* | > 579 (22) |  | 0.0048* |  |
|  |  |  |  |  |
| Wild-type N2 | > 535 (57) |  |  |  |
| JT528 *nrf-4(sa528)* | > 585 (43) |  | ns* |  |
| JT513 *nrf-5(sa513)* | > 692 (34) |  | < 0.0003* |  |
| JT525 *nrf-6(sa525)* | > 644 (49) |  | < 0.0003* |  |
|  |  |  |  |  |
| Wild-type N2 | 410 ± 60 (93) |  |  |  |
| OLS 53 *nrf-5 (sa513)* | 637 ± 113 (103) |  | <0.0001 |  |
|  |  |  |  |  |
| Wild-type N2 *EV(RNAi)* | 720 ± 108 (42) |  |  |  |
| Wild-type N2 *daf-16 (RNAi)* | 682 ± 127 (35) |  | 0.0436 |  |
| OLS51 *nrf-5 (sa513) EV(RNAi)* | 736 ± 120 (39) |  | 0.0301  <0.0001 |  |
| OLS51 *nrf-5 (sa513) daf-16 (RNAi)* | 698 ± 86 (70) |  | 0.0067 |  |
|  |  |  |  |  |
| Wild-type N2 *EV(RNAi)* | 635 ± 149 (45) |  |  |  |
| Wild-type N2 *ndg-4 (RNAi)* | 715 ± 127 (49) |  | 0.0258 |  |
| OLS51 *nrf-5 (sa513) EV(RNAi)* | 739 ± 144 (57) |  | 0.007  n.s. |  |
| OLS51 *nrf-5 (sa513) ndg-4 (RNAi)* | 716 ± 155 (42) |  | 0.0243 |  |
|  |  |  |  |  |
| Wild-type N2 OP50 | 336 ± 33 (41) |  |  |  |
| Wild-type N2 HB101 | > 459 (42) |  | <0.0001 |  |
| OLS51 *nrf-5 (sa513)* OP50 | > 448 (19) |  |  |  |
| OLS51 *nrf-5 (sa513)* HB101 | > 477 (32) |  | <0.0001 <0.0001 |  |
|  |  |  |  |  |
| Wild-type N2 OP50 | 403 ± 33 (43) |  |  |  |
| Wild-type N2 HB101 | 496 ± 66 (45) |  | <0.0001 |  |
| OLS51 *nrf-5 (sa513)* OP50 | > 598 (19) |  |  |  |
| OLS51 *nrf-5 (sa513)* HB101 | > 634 (21) |  | 0.0434 <0.0001 |  |
|  |  |  |  |  |

Table S1. Summary of longitudinal thermotolerance assays. For experiments on RNAi bacteria, P values correspond to comparison between worms fed RNAi bacteria with and without the indicated fragment in the vector and unless otherwise stated in the text worms were kept on RNAi for 2 generations to avoid maternal rescue. *After Bon-Ferroni correction.
