# Supplementary material for: Loss of NDG-4 extends lifespan and stress resistance in Caenorhabditis elegans
Source: Aging Cell. 2013 Nov 28;13(1):156–64. doi: 10.1111/acel.12165 (PMC3919970; doi:10.1111/acel.12165)
Supplement: Supplementary file 3 — Table S2 Summary of lifespan assays at 20 °C except experiment S1B#, which was at 25 °C. [file acel0013-0156-sd3.docx]

| Strain | Mean Survival Days ±SD (deaths recorded) | P-value  Log-rank test compared to controls | P-value  Log-rank test between specified strains |
| --- | --- | --- | --- |
| Wild-type N2 | 17.1 ± 2.4 (106) |  |  |
| CF1038 *daf-16(mu86)* | 15.1 ± 3.1 (82) | < 0.0001 |  |
| OLS11 *ndg-4(lb108)* | 29.1 ± 5.9 (104) | < 0.0001 |  |
| OLS15 *ndg-4(lb108); daf-16(mu86)* | 20.4 ± 3.2 (125) | < 0.0001 | < 0.0001* < 0.0001* |
|  |  |  |  |
| Wild-type N2 | 15.2 ± 3.4 (99) |  |  |
| CF1038 *daf-16(mu86)* | 14.8 ± 2.7 (159) |  |  |
| OLS11 *ndg-4(lb108)* | 27.7 ± 5.1 (108) |  | < 0.0001* < 0.0001* |
| OLS15 *ndg-4(lb108); daf-16(mu86)* | 19.9 ± 2.6 (109) |  |  |
|  |  |  |  |
| Wild-type N2 | 17.3 ± 4.8 (110) |  |  |
| JT529 *ndg-4(sa529)* | 28.4 ± 7.4 (95) | < 0.0001 |  |
|  |  |  |  |
| Wild-type N2 | 18.1 ± 4.9 (60) |  |  |
| JT529 *ndg-4(sa529)* | 25.3 ± 8.7 (45) | <0.0001 |  |
|  |  |  |  |
| Wild-type N2 | 18.3 ± 5.2 (96) |  |  |
| JT529 *ndg-4(sa529)* | 27.1 ± 6.9 (64) | < 0.0001 |  |
| OLS11 *ndg-4(lb108)* | 31.7 ± 6.2 (94) | < 0.0001 |  |
| F1 *ndg-4(lb108)*/*(sa529)* | 24.9 ± 5.4 ()93 | < 0.0001 |  |
|  |  |  |  |
| Wild-type N2 | 16.4 ± 2.7 (74) |  |  |
| OLS11 *ndg-4(lb108)* | 28.1 ± 5.3 (93) | < 0.0001 |  |
| RB754 *aak-2(ok524)* | 16.3 ± 2.6 (94) | ns |  |
| OLS17 *ndg-4; aak-2* | 20.9 ± 2.9 (108) | < 0.0001 | < 0.0001 <0.0001 |
|  |  |  |  |
| Wild-type N2 EV | 27.6 ± 4.9 (101) |  |  |
| Wild-type N2 *daf-2(RNAi)* | 43.6 ± 16 (124) | < 0.0001 |  |
| OLS11 *ndg-4(lb108)* EV | 33.0 ± 6.8 (129) | < 0.0001 |  |
| OLS11 *ndg-4(lb108) daf-2 (RNAi)* | 65.1 ± 18 (102) | < 0.0001 | < 0.0001 <0.0001 |
|  |  |  |  |
| Wild-type N2 EV | 21.5 ± 6.4 (142) |  |  |
| Wild-type N2 *daf-2(RNAi)* | 36.9 ± 18 (99) | < 0.0001* |  |
| OLS11 *ndg-4(lb108)* EV | 29.3 ± 7.2 (108) | < 0.0001* |  |
| OLS11 *ndg-4(lb108) daf-2 (RNAi)* | 44.2 ± 23.8 (126) | < 0.0001* | <0.0001 <0.0001 |
| OLS15 *ndg-4(lb108); daf-16(mu86)*  EV (RNAi) | 19.8 ± 5.1 (64) | 0.0185 |  |
| OLS15 *ndg-4(lb108); daf-16(mu86)*  *daf-2(RNAi)* | 19.4 ± 5.3 (84) | 0.0015 | ns |
|  |  |  |  |
| Wild-type N2 EV | 21.8 ± 5.0 (71) |  |  |
| Wild-type N2 *nhr-80(RNAi)* | 14.7 ± 1.7 (69) | < 0.0001 |  |
| Wild-type N2 *lipl-4(RNAi)* | 19.2 ± 4.0 (53) | 0.0004 |  |
| OLS11 *ndg-4(lb108) EV* | 29.6 ± 5.4 (120) |  |  |
| OLS11 *ndg-4(lb108) nhr-80(RNAi)* | 18.8 ± 2.9 (99) | < 0.0001 |  |
| OLS11 *ndg-4(lb108) lipl-4(RNAi)* | 28.6 ± 5.3 (107) | ns |  |
|  |  |  |  |
| Wild-type N2 EV | 18.5 ± 5.0 (70) |  |  |
| Wild-type N2 *nhr-80(RNAi)* | 13.5 ± 2.0 (68) | < 0.0001 |  |
| Wild-type N2 *lipl-4(RNAi)* | 19.7 ± 4.3 (46) | ns |  |
| OLS11 *ndg-4(lb108) EV* | 28.1 ± 5.2 (92) |  |  |
| OLS11 *ndg-4(lb108) nhr-80(RNAi)* | 18.7 ± 3.2 (99) | < 0.0001 |  |
| OLS11 *ndg-4(lb108) lipl-4(RNAi)* | 27.8 ± 5.8 (80) | ns |  |
|  |  |  |  |
| Wild-type N2 EV | 20.3 ± 5.0 (39) |  |  |
| Wild-type N2 *nhr-80(RNAi)* | 14.1 ± 1.1 (38) | < 0.0001 |  |
| Wild-type N2 *lipl-4(RNAi)* | 17.9 ± 4.5 (45) | ns |  |
| OLS11 *ndg-4(lb108) EV* | 26.5 ± 4.8 (50) |  |  |
| OLS11 *ndg-4(lb108) nhr-80(RNAi)* | 16.4 ± 2.4 (66) | < 0.0001 |  |
| OLS11 *ndg-4(lb108) lipl-4(RNAi)* | 26.4 ± 4.4 (23) | ns |  |
|  |  |  |  |
| Wild-type N2 | 15.6 ± 5.1 (73) |  |  |
| JT524 *nrf-1(sa524)* | 14.0 ± 2.8 (62) | ns* |  |
| JT528 *nrf-4(sa528)* | 14.6 ± 3.1 (121) | 0.0264* |  |
| JT513 *nrf-5(sa513)* | 20.3 ± 4.9 (133) | <0.0003* |  |
|  |  |  |  |
| Wild-type N2 | 19.8 ± 3.1 (107) |  |  |
| JT363 *nrf-3(sa363)* | 22.4 ± 3.9 (128) | <0.0003* |  |
| JT513 *nrf-5(sa513)* | 23.1 ± 4.1 (93) | <0.0003* |  |
| JT525 *nrf-6(sa525)* | 21.7 ± 2.7 (66) | <0.0003* |  |
|  |  |  |  |
| Wild-type N2 | 20.0 ± 5.1 (100) |  |  |
| JT363 *nrf-3(sa363)* | 21.6 ± 5.6 (92) | 0.0468* |  |
| JT513 *nrf-5(sa513)* | 24.6 ± 5.1 (102) | <0.0003* |  |
| JT525 *nrf-6(sa525)* | 22.3 ± 3.9 (46) | ns* |  |
|  |  |  |  |
| Wild-type N2 | 17.8 ± 3.3 (16) |  |  |
| *OLS51 nrf-5(sa513)* | 20.8 ± 3.0 (39) | <0.0001 |  |
|  |  |  |  |
| Wild-type N2 *EV(RNAi)* | 22.0 ± 4.9 (27) |  |  |
| OLS51 *nrf-5(sa513) EV(RNAi)* | 22.9 ± 5.7 (39) | ns |  |
|  |  |  |  |
| Wild-type N2 *EV(RNAi)* | 22.8 ± 6.1 (50) |  |  |
| OLS51 *nrf-5(sa513)* *EV(RNAi)* | 24.7 ± 6.4 (56) | Ns |  |
|  |  |  |  |
| Wild-type N2 HB101 | 19.8 ± 4.4 (46) |  |  |
| OLS51 *nrf-5(sa513)* HB101 | 22.7 ± 4.5 (83) | 0.002 |  |

Table S2. Summary of lifespan assays at 20 °C except experiment S1B^#^, which was at 25 °C. For experiments on RNAi bacteria, P values correspond to comparison between worms fed RNAi bacteria with and without (EV empty vector) the indicated fragment in the vector. In all other experiments worms were fed OP50. *After Bon-Ferroni correction. ns = not significant.
